# RETRACTION: HuR Promotes the Progression of Gastric Cancer through Mediating CDC5L Expression

**DOI:** 10.1155/dim/9838093

**Published:** 2025-03-31

**Authors:** Disease Markers

RETRACTION: Cui Jing, Nanjing Cao, Guochao Wang, Fuhua Wang, Bin Yang, Jian Wang, Yongqiang Lv, Yunqing Chen, and Feng Li. “HuR Promotes the Progression of Gastric Cancer through Mediating CDC5L Expression.” *Disease Markers* 2022, no. 1 (2022): 5141927. https://doi.org/10.1155/2022/5141927

The above article, published online on 12 March 2022 in Wiley Online Library (wileyonlinelibrary.com), has been retracted by John Wiley & Sons Ltd. due to concerns identified with Figures 2D and 5F as follows:

– Figure 2D – SGC-7901/si-Control and Figure 5F – MGC-803/miR-133bnc appear to be the same image rotated 90 degrees
– Figure 2D – SGC-7901/si-HuR and Figure 5F – MGC-803/miR-133b appear to be the same image rotated 90 degrees

The data reported in this article is therefore considered unreliable.

The authors were informed of the decision to retract but did not respond.

